# New alternative splicing BCR/ABL-OOF shows an oncogenic role by lack of inhibition of BCR GTPase activity and an increased of persistence of Rac activation in chronic myeloid leukemia

**DOI:** 10.18632/oncoscience.260

**Published:** 2015-11-11

**Authors:** Cristina Panuzzo, Gisella Volpe, Elisa Cibrario Rocchietti, Claudia Casnici, Katia Crotta, Sabrina Crivellaro, Giovanna Carrà, Roberta Lorenzatti, Barbara Peracino, Davide Torti, Alessandro Morotti, Maria Pilar Camacho-Leal, Paola Defilippi, Ornella Marelli, Giuseppe Saglio

**Affiliations:** ^1^ Department of Clinical and Biological Sciences, San Luigi Hospital, University of Turin, Orbassano, Turin, Italy; ^2^ Department of Oncology, San Luigi Hospital, University of Turin, Orbassano, Turin, Italy; ^3^ Department of Medical Biotechnologies and Translational Medicine, School of Medicine, University of Milan, Milan, Italy; ^4^ Department of Molecular Biotechnology and Health Sciences, University of Turin, Turin, Italy

**Keywords:** BCR-ABL, Chronic Myeloid Leukemia, Rac GTPase, alternative splicing

## Abstract

In Chronic Myeloid Leukemia 80% of patients present alternative splice variants involving *BCR* exons 1, 13 or 14 and ABL exon 4, with a consequent impairment in the reading frame of the *ABL* gene. Therefore BCR/ABL fusion proteins (BCR/ABL-OOF) are characterized by an in-frame *BCR* portion followed by an amino acids sequence arising from the out of frame (OOF) reading of the *ABL* gene. The product of this new transcript contains the characteristic BCR domains while lacking the COOH-terminal Rho GTPase GAP domain. The present work aims to characterize the protein functionality in terms of cytoskeleton (re-)modelling, adhesion and activation of canonical oncogenic signalling pathways. Here, we show that BCR/ABL-OOF has a peculiar endosomal localization which affects EGF receptor activation and turnover. Moreover, we demonstrate that BCR/ABL-OOF expression leads to aberrant cellular adhesion due to the activation of Rac GTPase, increase in cellular proliferation, migration and survival. When overexpressed in a BCR/ABL positive cell line, BCR/ABL-OOF induces hyperactivation of Rac signaling axis offering a therapeutic window for Rac-targeted therapy. Our data support a critical role of BCR/ABL-OOF in leukemogenesis and identify a subset of patients that may benefit from Rac-targeted therapies.

## INTRODUCTION

Chronic Myelogenous Leukemia (CML) is a myeloproliferative disorder characterized by the Philadelphia chromosome [1, 2], which originates from a reciprocal translocation involving the *BCR* gene on chromosome 22 and *c-ABL* on chromosome 9. The BCR/ABL protein product is a constitutively active oncogene (p210 BCR/ABL) which is necessary and sufficient for CML development [3, 4, 5]. Imatinib (IM) is the frontline therapy for CML patients, achieving an high rate of Major Molecular Response (MMR; defined as 3-log reduction in *BCR/ABL* transcript level from a standardized baseline value) and Complete Molecular Response (CMR; defined as at least 4-log reduction corresponding to undetectable *BCR/ABL* transcript by real-time reverse transcription polymerase chain reaction) [6, 7] which might represent an ‘operational cure’ as well as a pre-requisite for IM discontinuation [8].

Still, there are a number of patients with p210 BCR/ABL carrying kinase mutations that may contribute to IM resistance. The second generation kinase inhibitors Dasatinib and Nilotinib have been developed and are currently used in IM non-responder CML patients [9, 10], even though the chance to select a clone resistant to both drugs is still high [11].

In order to seek alternative pharmacological approaches (i.e. druggable molecules), we focused on the extra components downstream of BCR/ABL which may represent additional molecular targets for (IM) resistant patients.

As reported in our previous reports [12], we identified a new alternative splice variant derived from *BCR/ABL* transcript in which the fusion involved the exons 1-13 or 14 of *BCR* and exon 4 (in spite of exon 2) of *c-ABL*. This alternative splicing induces a change in the reading frame of *ABL* portion generating an early STOP codon in the middle of *c-ABL* exon 5. The latter results in the production of a fusion protein defined by an initial (and correctly read) BCR portion attached to a sequence of amino acids arising from the out of frame (OOF), leading to the *c-ABL* exon 4 and 5 gene sequence.

It is worth mentioning that this protein is largely studied for its attractive immunological function [13, 14, 15]. In order to investigate a possible Bcr/Abl-OOF oncogenic role we focused our attention on its peculiar structure generated as a consequence of the alternative splicing. The *BCR/ABL*-OOF product gene is a 120 kDa protein which lacks the BCR COOH-terminal Rac GAP domain [16], which is fundamental to balance cytoskeleton dynamics trough the regulation of the Rho-like GTPases activity [17, 18].

Rho GTPases have been previously described for their important roles in Bcr/Abl-mediated transformation as downstream components, with a specific involvement for Rac GTPases [19, 20, 21]. Interestingly, in a leukemia murine model, the deficiency of Rac1 and Rac2 leads to a significant attenuation of the malignant phenotype. Furthermore a Rac-specific small molecule inhibitor (NSC23766) was able to reduce the leukemic burden *in vivo* in NOD/SCID mice transplanted with chronic-phase CML CD34+ cells [22].

Thus, to determine the role of the BCR/ABL-OOF, we analyzed the effects of the protein in terms of cytoskeleton (re-)modelling, adhesion, activation of Rho GTPases and oncogenic pathways. Altogether the current study identifies a new role for the Bcr/Abl transcript variant protein product and thus it may suggest its new potential as therapeutic target in CML.

## RESULTS

### BCR/ABL-OOF localizes predominantly in microsomes of HeLa cells

To study the proper BCR/ABL-OOF protein localization, we used the expression vector pCDNA 3.1 *BCR/ABL*-OOF, generated by our laboratory as previously described [13].

**Figure 1:**
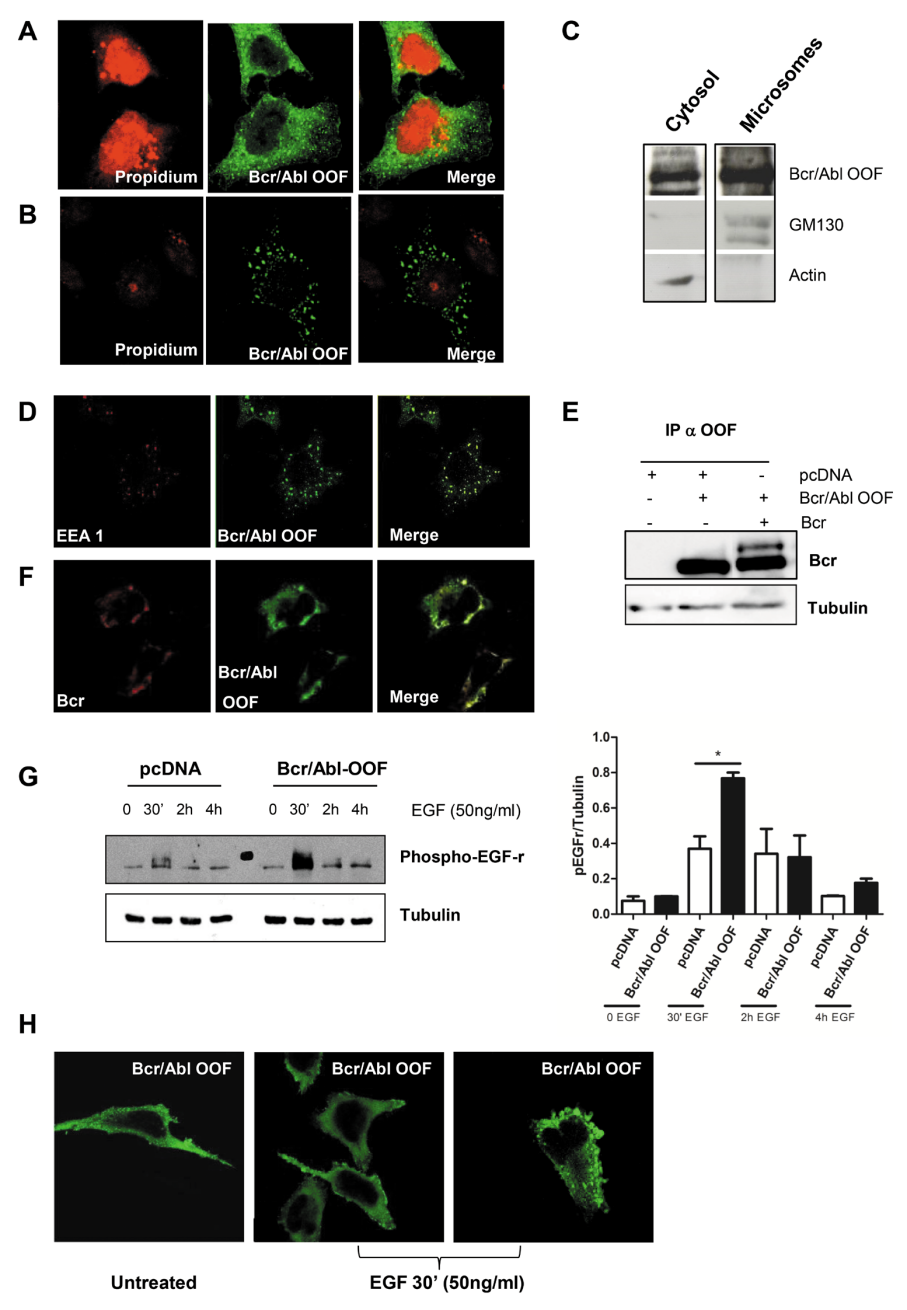
BCR/ABL-OOF localized predominantly in endosomes of HeLa cells where it interacts with p160 BCR Immunofluorescence assay using Rabbit anti OOF policlonal antibody on BCR/ABL-OOF HeLa transfected cells before (A) and after (B) the treatment with PIPES and 0,1% Saponin C) Western blot analysis of cytosolic and microsomal fractions of BCR/ABL-OOF transfected HeLa cells. Protein extracts were examined for expression of Bcr/Abl-OOF by immunoblotting with polyclonal Rabbit anti OOF. Actin and GM130 expression, were detected, using specific antibodies, as markers respectively of the cytosolic and microsomal compartment. D) Immunofluorescence with antibody anti EEA1 (red signal) and BCR/ABL-OOF (green signal) on Hela cells trasfected with BCR/ABL-OOF.E) Immunoprecipitation of protein extracts from HeLa cells co-expressing exogenous BCR/ABL-OOF and p160 BCR using rabbit anti-OOF antibody followed by immunoblotting with anti Bcr. F) Immunofluorescence with antibody anti BCR (red signal) and BCR/ABL-OOF (green signal) on Hela cells cotrasfected with pcDNA *BCR/ABL-OOF* and p160 *BCR*. G) Analysis of EGF-receptor activation in BCR/ABL-OOF Hela cells in term of phosphorylation by western blot after EGF ligand stimulation (50 ng/ml at different time point: 0, 30’, 1h, 2h and 4h). H) Localization of BCR/ABL-OOF by immunofluorescence after EGF ligand stimulation (50 ng/ml at different time point: 0, 5’, 10’, 30’ and 60’. Figure 1G is representative of 30’ of EGF stimulation).

Hela cells expressing BCR/ABL-OOF protein were subjected to immunofluorescence and western blot assay 48 h post-transfection. The immunofluorescence assay revealed that BCR/ABL-OOF presents a peculiar vesicular and cytoplasmatic localization (Figure 1A). The same assay performed with PIPES 0,1% saponin showed that BCR/ABL-OOF maintained a strong signal inside the vesicular compartment. Altogether these results supported our hypothesis that BCR/ABL-OOF is linked to the cytoskeletal structure (Figure 1B).

HeLa BCR/ABL-OOF expressing cells were lysed with a previous described protocol [23] which allow to preserve different subcellular compartments. We focused on the microsomal fraction containing a mixture of many organelles, such as Golgi, ER and intracellular vesicles. As shown in Figure 1C, BCR/ABL-OOF is expressed in large amount in the vesicular compartment, confirming the results obtained by immunofluorescence.

### BCR/ABL-OOF is localized in endosomal vesicles and interacts with p160 BCR

To investigate which type of vesicles were associated to BCR/ABL-OOF, immunofluorescence analysis of BCR/ABL-OOF and of a known marker of early endosomal vesicles EEA1 was performed after PIPES 0,1% saponin treatment. BCR/ABL-OOF protein exhibited a similar staining pattern localization in endosomal vesicles where EEA1 is physiologically located (Figure 1D). The same result was confirmed on cells co-trasfected with BCR/ABL-OOF and p160 BCR, which is localized in the endosomal compartment [24, 25]. It is well known that BCR is a component of mammalian ESCRT complexes and it plays an important role in proteins sorting during endosomal trafficking by interaction with TSG101. Because the region involved in this interaction is lost in BCR/ABL-OOF, we investigated whether a direct interaction between p160 BCR and Bcr/Abl-OOF could be responsible for its localization. Indeed, Immunoprecipitation analysis show that BCR/ABL-OOF and p160 BCR co-immunoprecipitate (Figure 1E). Immunofluorescence analysis showed a co-localization of BCR and BCR/ABL-OOF in endosomal vescicles (Figure 1F), thus confirming previous results.

Next, given the importance of endosomial p160 BCR in the physiological turnover and recycling of EGF receptor [24] we decide to investigate whether BCR/ABL-OOF was also involved. In order to evaluate this possibility, we analyzed EGF receptor activation levels in the presence of BCR/ABL-OOF protein upon EGF ligand stimuli. By western blot analysis, we observed an increased in phosphorylation levels of EGF receptor compared to cells transfected with empty vector (Figure 1G). This result is also supported by immunofluorescence analysis where there is an increase in the submembrane localization of BCR/ABL-OOF at 30 minutes of EGF ligand stimulation (Figure 1H). This result suggests that BCR/ABL-OOF may cooperate in endosome-mediated receptor internalization.

### BCR/ABL-OOF expression leads to Rac activation

To understand whether the absence of GAP domain as a consequence of *BCR/ABL* alternative splicing could confer some peculiarity to BCR/ABL-OOF protein, we investigated the status of Rho family proteins in HeLa *BCR/ABL*-OOF expressing cells in comparison to cells transfected with the active forms of Rho or Rac.

**Figure 2:**
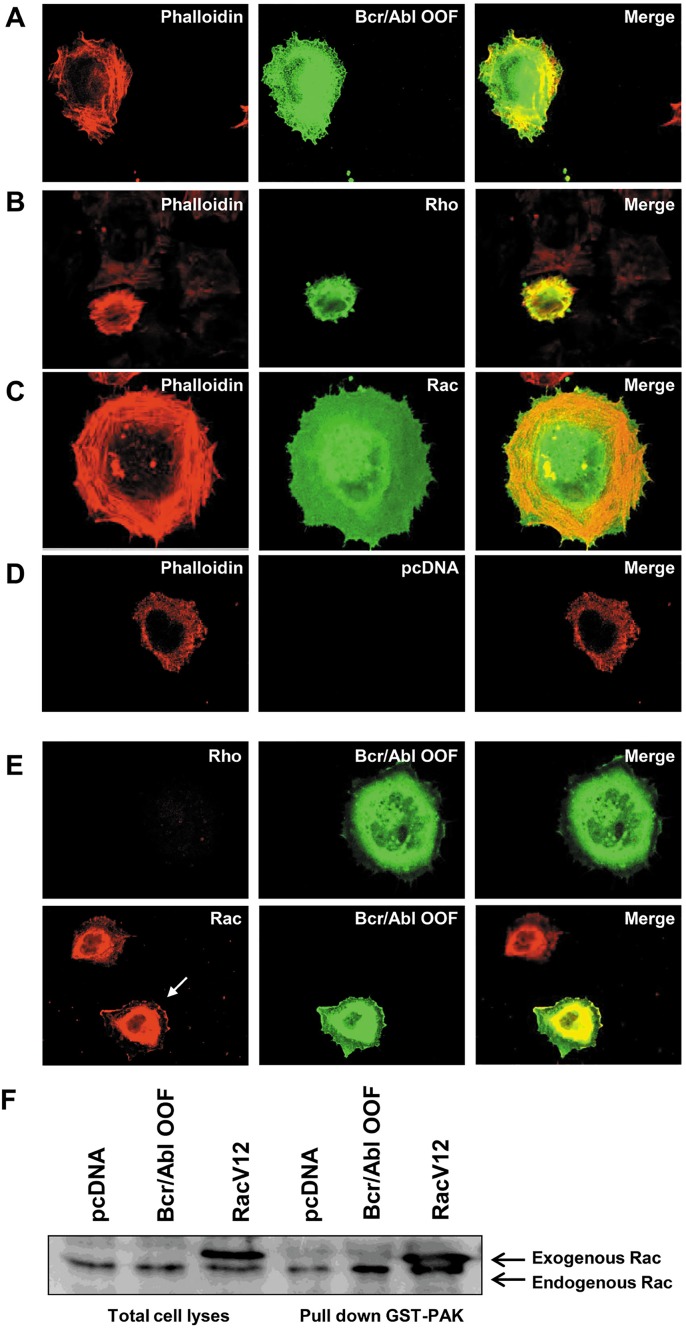
Rac is activated in cells transfected with pcDNA BCR/ABL-OOF HeLa cells were respectively transfected with different constructs, pcDNA Bcr/Abl-OOF (A), pEXV-mycRhoV1 (B), pEXV-mycRacV12 (C) and empty pcDNA (D). Immunofluorescence was performed after one hour adhesion on fibronectin coated coverslips by using the specific antibody rabbit anti-OOF (A), mouse anti-Rho (B) and mouse anti-Rac (C) subsequently evidenced by FITC secondary antibody. Red phalloidin was used to take evidence of cytoskeleton structure. E) cells transfected with pcDNA *BCR/ABL-OOF* were used for immunofluorescence assay performed with rabbit anti-OOF (green signal) and respectively with mouse anti-Rac or mouse anti-Rho (red signal) after one hour adhesion on fibronectin coated coverslips. Rac partial membrane or submembrane localization suggested its activation form. F) Lysates derived from HeLa cells transfected with empty pcDNA, *BCR/ABL-OOF* or pEXV-mycRacV12 were incubated with GST PAK-CD for the pull down assay and subsequently processed by western blot.. 15 μg of total lysate was shown as control of equal quantity of lysate in the different fractions.

Since cell adhesion can be modified by the activation pattern of Rho-like GTPases through an inside-out mechanism we performed an adhesion assay as described in materials and methods. HeLa cells expressing BCR/ABL-OOF showed significant modifications in spreading to fibronectin respect to control cells transfected with empty vector. Their morphological pattern was quite similar to that of HeLa cells transfected with the active form of Rac, while differing from those transfected with the active form of Rho (Figure 2A, 2B, 2C, 2D).

We next decided to perform immunofluorescence analysis of HeLa BCR/ABL-OOF expressing cells by using a dual staining with anti-OOF and anti-Rho or anti-Rac antibodies respectively. We observed that while endogenous Rho remained totally diffused inside the cell, endogenous Rac had a peculiar distribution under the cell membrane which corresponded to Rho GTPases activated form localization [26] (Figure 2E). These results suggest that Rac activation may be responsible for mediating BCR/ABL-OOF cell morphology changes during cell adhesion to fibronectin.

The activation of Rac was also confirmed by a pulldown assay using Rac GTPase binding domain as GST fusion protein bound to glutathione-sepharose beads (GST PAK-CD). This fusion protein was able to trap a significant proportion of GTP-bound Rac form in lysates coming from BCR/ABL-OOF HeLa cells in the same manner as cells transfected with the active form of Rac (Rac V12) thus supporting the presence of Rac in the active form (Figure 2F).

### BCR/ABL-OOF induces cellular proliferation, migration and survival

**Figure 3:**
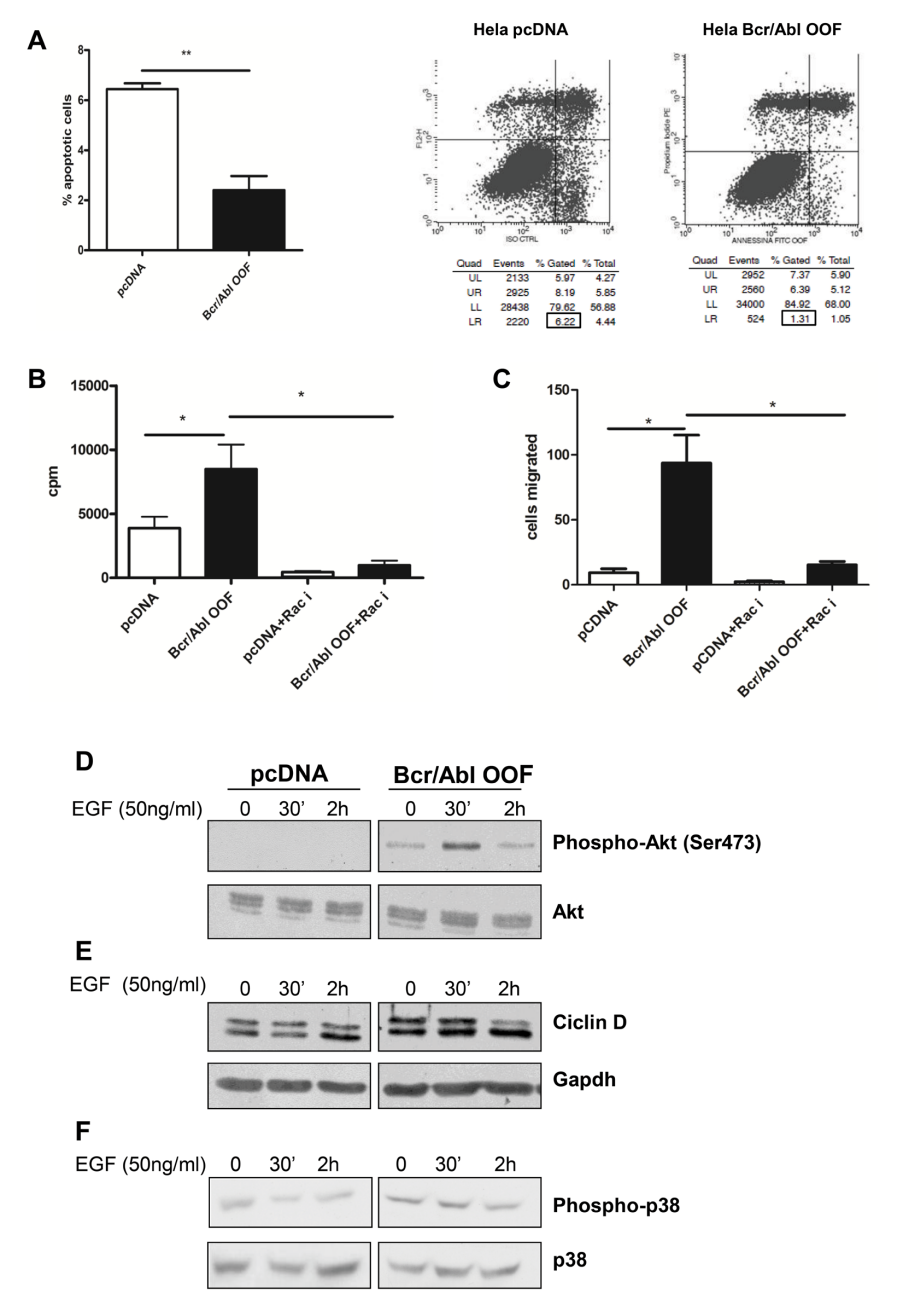
BCR/ABL-OOF induces cellular proliferation, migration and survival A) Apoptosis rate on HeLa BCR/ABL-OOF or empty vector was analyzed by flow cytometry analysis with FITC Annexin V antibody and PI Propidium cell staining. B) 1 μCi/ml of timidine (^3^H) was incubated for 24 hours during proliferation in cells transfected with empty vector or with *BCR/ABL-OOF* in basal condition or after incubation with Rac inhibitor NSC23766. β counter measure is expressed as cpm (counts per minute). C) Migration assay was performed with 1 × 104 cells by using 8μm pore size transwell. Cells migrated after 22 hours were counted by stained with May Grunwald Giemsa staining. D) Forty eight hours after transfection, HeLa cells were starved for 12 hours and stimulated with EGF ligand (50 ng/ml) for different time point (0, 30, 120 minutes). Lysates were collected and they were examined by western blot for expression of pAKT (D), Cyclin D (E) and pp38 (F). Akt, GAPDH and p38 were used as loading control. The quantification was performed by ImageJ program.

On the basis of the latter results, we were interested to investigate a possible involvement of BCR/ABL-OOF in the activation of canonical oncogenic signalling pathways. For this purpose, HeLa cells were trasfected with pcDNA *BCR/ABL-OOF* and 48 h post-transfection cells were selected with G418 for 14 days to enrich the amount of positively transfected cells. Apoptosis assay illustrates that BCR/ABL-OOF cells have a lower apoptotic rate than transfected cells with empty pcDNA (normal control), with 62% reduction of apoptosis (p = 0,002) (Figure 3A). Proliferation assay was performed through evaluation of 3H incorporated in proliferating cells. These results suggest that BCR/ABL-OOF cells proliferate more compared with pcDNA control cells (2 fold of induction, (Figure 3B). In addition, migration assay performed by using 20% serum as stimuli shown an increased capacity of BCR/ABL-OOF cells to migrate compared to control cells (Figure 3C).

In order to confirm the importance of Rac activation in the behaviour of BCR/ABL-OOF positive cells we decided to treat these cells with a specific Rac inhibitor, NSC23766. [21]. In accordance with previous results, we found both the proliferation and migration of BCR/ABL-OOF HeLa expressing cells strongly reduced. In addition, we observed an increased in apoptosis. Altogether these results suggest that Rac signalling could be a major component in BCR/ABL-OOF-mediate activity (Figure 3B and 3C).

In order to evaluate whether BCR/ABL-OOF expression could affect canonical oncogenic signalling pathways, we next analysed by western blot possible differences in Akt, Cyclin D and p38 expression. (Figure 3D, 3E and 3F).

Interestingly, we found that basal phospho-Akt (Ser473) levels were already significantly increased in cells transfected with BCR/ABL-OOF, with the highest expression at 30 minutes of EGF stimulation (p< 0.001) (Figure 3D); the latter result may correlate with the basal reduction of apoptosis observed in these cells.

Cyclin D was analysed as positive activator of DNA synthesis and, as consequence, of cells proliferation; our data demonstrated an increased cyclin D level in BCR/ABL-OOF cells both in basal condition and under EGF stimulation, confirming the results obtained in the proliferation assay (Figure 3E).

Finally, the stress-activated p38 mitogen-activated protein (MAP) kinases was also analysed due to its strong Rac regulation; it is well described that constitutively active forms of Rac leads to activation of p38 by the action of p21-activated Kinase 1 (Pak1) as mediator [27]. Interestingly, under basal conditions, p38 activation is already high in cells transfected with pcDNA *BCR/ABL-OOF* even in absence of growth factor stimuli, confirming the involvement of Rac activity as a result of BCR/ABL-OOF protein expression in this cells (Figure 3F).

Altogether these results suggest BCR/ABL-OOF as an important player during cell proliferation, survival and apoptosis in chronic myeloid leukaemia.

### BCR/ABL-OOF can cooperate with BCR/ABL oncogenicity

**Figure 4:**
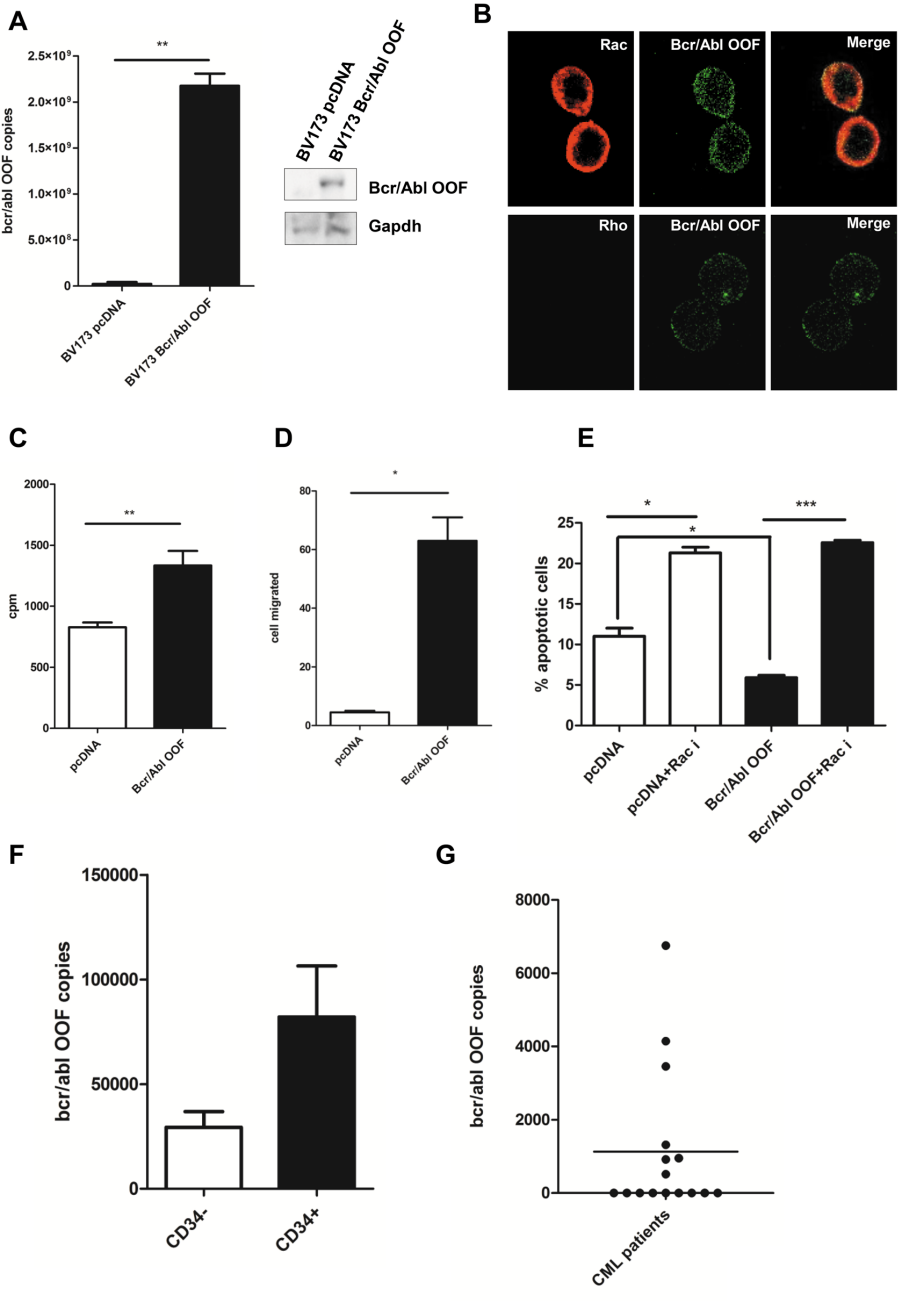
BCR/ABL-OOF can cooperate with BCR/ABL oncogenicity A) RTQ-PCR assay and Western blot showing *BCR/ABL-OOF* transcript and protein levels in BV173 Ph+ cell line transfected respectively with empty pcDNA and pcDNA *BCR/ABLOOF* B) Immunofluorescence assay performed on BV173 cells line transfected with pcDNA *BCR/ABL-OOF* using rabbit anti-OOF (green signal) and respectively mouse anti-Rac or mouse anti-Rho (red signal) antibodies after twenty-four hour of adhesion on fibronectin coated coverslips. C) Proliferation assay and D) migration assay performed on BV173 cell line transfected respectively with empty pcDNA and pcDNA BCR/ABL-OOF. E) Apoptosis assay on BV173 BCR/ABL-OOF positive after incubation with Rac inhibitor F) RTQ-PCR assay showing copies number of *BCR/ABL-OOF* transcript in CD34+ cell fraction coming from the bone marrow of 7 CML patients at diagnosis G) Representative analysis of the copies number of *BCR/ABL-OOF* transcript after three months of Imatinib treatment.

As we have previously reported, *BCR/ABL-OOF* is an aberrant transcript that coexists with full length *BCR-ABL* transcript [12]. We therefore sought to investigate whether BCR/ABL-OOF and BCR/ABL could synergize in leukemia cells. To this aim, we transfected BCR/ABL-OOF in BV173 Ph+, hematopoietic cells which are characterized by a very low expression of BCR/ABL-OOF protein. After confirming post-transfection BCR/ABL-OOF expression levels by western blot and mRNA analysis (Figure 4A) Rac GTPase activity was evaluated upon fibronectin cell adhesion (Figure 4B). Under this condition we observed a strong Rac GTPase activation in BV173 BCR/ABL-OOF positive cell line meanwhile endogenous Rho pathway seemed to be not activated. A strong significant increased in cell proliferation (p = 0,008) and migration (p = 0,02) were observed in BCR/ABL-OOF positive samples (Figure 4C and 4D). Interestingly, BV173 BCR/ABL-OOF cells are more sensitive to Rac inhibition, confirming the Rac pathway addiction in BCR/ABL-OOF cells (Figure 4E). Given the high levels of Rac GTPases in CD34+ of CML patients and its importance as a key stimulator of ROS production [28] we decided to analyse by RT-Q-PCR the copy number of *BCR/ABL-OOF* transcript on CD34+ positive cells derived from the bone marrow of 7 CML patients. We observed a trend to increased mRNA *BCR/ABL-OOF* transcript expression compared to the CD34- fraction (Figure 4F), confirming the importance of BCR/ABL-OOF in the activation of the Rac pathway, especially in the more primitive leukemic compartment where it is highly expressed.

In order to find a correlation with the *in vitro* results, we next analyzed the expression levels of *BCR/ABL-OOF* in 16 patients after 3 months of imatinib treatment. In three patients, our analysis showed a slow reduction of *BCR/ABL-OOF* transcript levels after three months of imatinib therapy. Interestingly, these patients display a worst response to Imatinb treatment (Figure 4G). These analyses raised the possibility that BCR/ABL-OOF cooperates with the oncogenic BCR/ABL pathway, suggesting a new attractive alternative to treat patients with a suboptimal response.

## DISCUSSION

Alternative splicing (AS) is an essential biological process which drives proteome diversity. Besides AS role in many physiological processes [29], it represents an intriguing player also in cancer pathogenesis through a variety of mechanisms. Foremost, AS itself may be the result of mutations in genes that are involved in the regulation of the spliceosome, leading to the aberrant expression of several genes [30]. More recently, even in the absence of spliceosome gene mutations, the alternative splicing of specific genes has also been reported to bring important biological consequences. In this context, AS has been previously postulated to have potential therapeutic implications [30].

For instance, our previous results have shown that *BCR/ABL* alternative splicing isoforms lead to the expression of aberrant proteins [12]. Importantly, these proteins display immunological properties, thus offering a therapeutic window [12].

Here, we demonstrate an additional role for BCR/ABL alternative splicing isoforms. In particular, we show that alternative proteins may have a potential role during tumorigenesis. We provide evidences demonstrating that BCR/ABL-OOF protein is able to increase cellular proliferation and migration and to inhibit apoptosis. Although tyrosine-kinase activity of BCR/ABL is essential for CML pathogenesis, additional BCR/ABL functions along with hematopoietic stem/progenitor cells and tumour microenvironment biology are likely to contribute to the disease development and maintenance.

It is well described the p210 capability to induce an altered cell adhesion [31]; our data indeed demonstrate that BCR/ABL-OOF seemed to have an analog role and we hypothesizes that it may cooperate with BCR/ABL in massive cytoskeleton reorganization processes.

Herein we clearly demonstrate the ability of BCR/ABL-OOF to activate Rac. In particular, higher BCR/ABL-OOF expression levels were found in most undifferentiated hematopoietic CD34+ cells compared to CD34-cell population and indicate Rac as a new therapeutical target to block in order to inhibit leukemia stem cells.

Final point is the peculiar localization of Bcr/Abl-OOF. This event may help Bcr/Abl-OOF to exert its GEF activity on the Rho GTPases, which function in the control of vesicular trafficking is well known [32, 33]. Moreover it could be implicated on the EGF receptor turnover, a process deregulated in many solid tumors [34]. Recent research showed the ability of an insect extracts (E. sinensis Walker) to inhibit the proliferation of leukemia K562 by inhibiting EGF secreting and blocking the EGFR signaling pathway, suggesting a relevance of EGFR in human hematological diseases [35]. Another study [36] showed the EGF receptor ability to induce activation of non-canonical MAPK (p38, MEK/ERK) and PI3K pathways [37]. For these reasons targeting EGF and its direct activators could be a valuable alternative to inhibit tumor progression.

These interesting results will be prospectively tested in patient cells (i.e. *ex vivo*) in order to confirm all the evidence obtained in *in vitro* cell line model. The fact that patients with slow reduction of both BCR/ABL and BCR/ABL-OOF shown a suboptimal response could lead us to the conclusion that the lack of GAP domain in BCR/ABL-OOF could induce an increased of persistence of Rac activation and increase cell tumorigenesis.

All together our data suggest that BCR/ABL-OOF is actively involved in the BCR/ABL signalling due to its specific oncogenic properties and it could provide additional targets for developing new drugs in order to eradicate CML stem cells. This speculation could have tremendous consequences from the therapeutic standpoint. Although additional investigations are necessary to better establish the overall impact of AS transcript in cancer pathogenesis, this work suggests that the cancer trascriptional machinery could act favoring the expression of many oncogenic isoforms which could be responsible of different sensitivity to therapeutic agents (i.e. TKI) [38].

## MATERIALS AND METHODS

### Plasmids

The pcDNA 3.1 *BCR/ABL-OOF* construction was previously described by Casnici *et al.* 2011 [14]. The PAULO-*BCR* p160 was kindly provided by M. Ruthardt (16); the pcDNA 3.1 BCR/ABL was generated by subcloning the 7kb EcoRI-*BCR/ABL* cDNA contained in pGD210, kindly provided by GQ. Daley [39], in pcDNA 3.1. pGEX PAK-CD, pEXV-mycRhoV1, pEXV-mycRacV12 were kindly provided by P. Defilippi (MBC, University of Turin).

### Human CML samples

16 Primary CML bone marrow samples were collected at diagnosis after informed consent. CD34 positive cells were isolated from 7 bone marrow aspirate accordingly to the Miltenyi Biotec protocol for CD34 purification (Miltenyi Biotec, #130-094-531). The amount of the alternative transcripts in patients RNA at diagnosis and after three months of therapy was evaluated as previously described [12].

Cell culture, transfection assay and pharmacological treatments: HeLa cells line was maintained in RPMI 1640 (Euroclone) supplemented with 10% heat-inactivated fetal bovine serum (FBS, Sigma Aldrich) and 2 mM glutamine (Euroclone) at 37°C in a 5% CO_2_ humified atmosphere, while BV173 (ACC 20, DSMZ) were maintained in RPMI 20% FBS. Transient transfections were performed using Fugene HD transfection reagent (Roche Diagnostic S.p.A, Milan, Italy) according to the manufacturer's instructions. To enrich the percentage of transfected cells, two days after the transfection stable clones were selected with G418 (100μg/ml) and then expanded to perform functional assays. For functional experiments 50 ng/ml Epidermal Growth Factor ligand (EGF; E9644, Sigma-Aldrich, St. Louis, MO) was added to the medium for different times points (0, 30 and 120 minutes) 24 hours post transfection. 80μM of Rac inhibitor NSC23766 (Calbiochem, EMD Millipore Corporation, Billerica, MA, USA) was used for 24 hours to inactivate Rac activity in HeLa cells.

Antibodies used in western blotting, immunoprecipitation and immunofluorescence assay: western blot analysis were performed according to widely used protocols using antibodies directed against Bcr, Ciclin D, (Santa Cruz Biotecnology, Dallas, Texas, USA), OOF (α BCR/ABL-OOF) (rabbit polyclonal of our production), phosphorylated EGF receptor, total and phosphorylated Akt (Cell Signalling technology, Danvers, MA) total and phosphorylated p38 (Cell Signalling technology, Danvers, MA). Mouse monoclonal GM130 (BD Trasduction Laboratories, BD Biosciences, California, USA), Actin and GAPDH (Santa Cruz Biotecnology, Dallas, Texas, USA) antibodies were also used as loading control proteins (Santa Cruz Biotecnology, Dallas, Texas, USA).

Immunofluorescence assay was performed with Rac (EMD Millipore Corporation, Billerica, MA, USA), Rho (Santa Cruz Biotecnology, Dallas, Texas, USA), Bcr (Santa Cruz Biotecnology, Dallas, Texas, USA), EEA1 and rabbit polyclonal anti-OOF.

### Immunofluorescence assay

HeLa cells plated on glass coverslips were fixed in 4% PFA for 10 min After cell permeabilization with 0,5% triton for 5 min, cells were blocked for 45 min with PBS 10% BSA. Cells were then incubated for 2 hours with the specific primary antibody, and the immunocomplex was detected by 30 min incubation with secondary antibody (Alexa Fluor 488 or 543, Life Technologies Corporation). Phalloidin (Alexa Fluor 568, Life Technologies Corporation) was also used to analyse the cytoskeleton structure by incubation for 30 min after secondary antibody incubation. Cells were visualized under a Leica Gmbh fluorescence microscope using Qfluo software (Leica Microsystem) and confocal scanning microscope (LSM 5110; Carl Zeiss Microlmaging Inc., 63X objective). To analyze the vesicular compartment, before fixation cells were treated with a solution containing PIPES (piperazine-N,N′-bis(2-ethanesulfonic acid)), 0,1% Saponin. This solution lead to the formation of micro pores on the cell membrane. Under this experimental condition all cytoplasmatic components are eliminated and only the cytoskeleton and the organelle structure remain linked to the cell

### Cell lysis, immunoprecipitation and western blot assay

Total cell extraction was performed using TNE buffer (50 mM Tris pH 8,8, 2 mM EDTA, 1% NP-40) supplemented with protease and phosphatise inhibitors (1 mM Na_3_VO_4_, 1 mM PMSF, 2μg/ml leupeptin, 2μg/ml aprotinin, 2μg/ml pepstatin). Protein extracts were clarified by centrifugation at 12000g for 15 min at 4°C and subsequently quantified with Bio-Rad Protein Assay (BioRad laboratories, CA, USA). For immunoprecipitation experiments 500 μg proteins extracts were pre-cleared with agarose-conjugated normal IgG (SantaCruz Biotechnology, Dallas, Texas, USA) for 1 hour at 4°C and then precipitated overnight with 1μg of the appropriate antibody along with protein A-agarose beads. The immunoprecipitate and protein samples were then resolved by SDS-PAGE and subsequently transferred onto nitrocellulose filter.

Immunoblots were then probed overnight at 4°C with specific antibodies in PBS 0,1% tween, 1% BSA and protein detection was performed by using peroxidise-conjugated secondary antibodies and chemiluminescent enhanced chemiluminescence reagent.

### Mixed microsomal fractions extraction

GST Pulldown Assay to evaluate Rac To isolate cytosol from microsomal fractions, and a mixture of different organelles (Golgi, endoplasmic reticulum and intracellular vesicles), a previously described protocol was used [23]. Briefly, the cells were resuspended into 250-STMDPS buffer (250 mM sucrose, 50 mM Tris-Hcl (pH 7,4), 5 mM MgCl_2_, DTT 1 mM, protease and phosphatase inhibitors), homogenized for 2 min and subsequently centrifuged at 800g for 15 min. To obtain a cleaner preparation, the supernatant was centrifuged a second time and then collected for the isolation of mitochondria, cytosol and microsomes. To discard the mitochondria contamination, the supernatant collected was subjected to another centrifuged at 6,000g for 15 min. To separate the cytosol from microsomal fraction supernatant was centrifuged in a swing-bucket ultracentrifuge at 100,000g for 1 hr. Once the supernatant was collected as pure cytosol, the remaining pellet was solubilised in ME buffer (20 mM Tris-HCl (pH 7,8), 0,4M NaCl, 15% glycerol, 1 mM DTT, 1,5% Triton-X-100, protease and phosphatase inhibitors) and centrifuged at 9,000g for 30 min to extract microsomal proteins. Finally the different fractions were frozen for analysis at a later date by western blot using polyclonal anti OOF antibody and specific antibodies of cytosolic marker compartment (anti Actin, Santa Cruz Biotechnology) and microsomal (anti GM130, BD Transduction Laboratories, BD Biosciences).

activity: pGEX PAK-CD was expressed in TOP F10 cells by induction with isopropyl-L-thio-B-D-galactopyranoside (IPTG) 0,1 mM according manufacturer's instructions. Briefly, bacteria were pelleted, resuspended in lysis buffer and sonicated. After centrifugation, the clarified cell lysate containing the fusion protein GST-PAK was incubated with GST-fusion protein beads for 90 min at 4°C. The beads linked with GST-PAK were washed 4 times with PBS 0,5% triton to remove the unbound proteins and subsequently incubated for 1 hour with GST FISH buffer lysates (5% glycerol, 50 mM Tris pH 7,4, 100 mM NaCl, 2 mM MgCl2) from HeLa cells transfected with pCDNA 3.1 *BCR/ABL-OOF*, pcDNA 3.1 *RACV12* as positive control, respectively and empty pcDNA 3.1 as negative control. Beads-bound complexes were washed four times with FISH buffer and boiled in Laemmli sample buffer. The samples were fractionated in 15% SDS-PAGE and subsequently transferred to a nitrocellulose membrane for western blot analysis.

### Apoptosis assay

Apoptosis rate was analysed by flow cytometry analysis, using Annexin V Kit reagent (Immunostep Research). Cells transfected with empty pCDNA3.1 and pcDNA 3.1 BCR/ABL-OOF were detached and incubated with anti Annexin V antibody and red propidium for 15 min, respectively. Cells were washed with PBS and then analysed by flow cytometry in order to quantify the apoptosis rate under different cells conditions.

### Proliferation assay

Forty-eight hours after transfection, empty pCDNA 3.1, pcDNA 3.1 *BCR/ABL-OOF* and pcDNA 3.1 *BCR/ABL* cells were starved for 12 hours and 1 μCi/ml of thymidine (GE Healthcare, Piscataway Township, NJ, USA) was added with or without 80 μM of Rac inhibitor NSC23766. Thymidine incorporation was evaluated after 24 hours; cells were washed 2 times with cold PBS and fixed with 5% trichloroacetic (TCA) for 30 min, respectively. After washing, NaOH 1M was added for 20 min followed by adding HCl 2M to stop the reaction. The correct quantity of Thymidine incorporated was measured by a β-counter instrument.

### Spreading assay

HeLa cells transfected with respective constructs were detached with PBS 5 mM EDTA after overnight starvation and were plated on 10μg/ml fibronectin-coated glass coverslips (Marlenfeld GmbH&Co.KG, Germany) with RPMI without serum. The well plate was placed into an incubator at different time conditions and rapidly fixed with 4% PFA. Immunofluorescence assay was then performed using the specific antibodies and phalloidin to stain actin filamentsstaining.

### Migration assay

Transwell filters were used (8μm pore size plate, Corning Incorporated, Corning, NY, USA) to analyse cell migration of transfected HeLa cells. Cells were detached from the cell plate with PBS 5 mM EDTA and 1 × 10^4^ cells were seeded in the upper layer of a transwell after an overnight starvation. The transwell membrane was coated with fibronectin 10 mg/ml at 4°C overnight to facilitate the cell's adherence. A specific stimulation (RPMI+FBS 20%) was placed below the cell permeable membrane. Cells were incubated for 22 hours at 37°C, 5% CO_2_ Transwell filters were washed three times with PBS and migrating cells were visualized and counted by May Grunwald Giemsa staining (Diapath, Martinengo, Italy).

### Statistics

Two-sided Student's t test was calculated using GraphPad Prism software. P values < 0.05 were considered statistically significant.
